# Low-dose capecitabine adjuvant chemotherapy in elderly stage II/III colorectal cancer patients (LC-ACEC): study protocol for a randomized controlled trial

**DOI:** 10.1186/s13063-015-0753-7

**Published:** 2015-05-29

**Authors:** Yazhou He, Ping Liu, Yuanchuan Zhang, Xiangbing Deng, Wenjian Meng, Mingtian Wei, Tinghan Yang, Ziqiang Wang, Meng Qiu

**Affiliations:** Department of Gastrointestinal Surgery, West China Hospital, Sichuan University, # 37 Guo Xue Alley, Chengdu, Sichuan 610041 PR. China; West China School of Medicine, West China Hospital, Sichuan University, # 37 Guo Xue Alley, Chengdu, Sichuan 610041 PR. China; Colorectal Cancer Clinical Research Center, The Third Affiliated Hospital of Kunming Medical University, # 519 Kunzhou Road, Kunming, Yunnan 650118 PR. China; Department of Abdominal Cancer, Cancer Center, West China Hospital, Sichuan University, # 37 Guo Xue Alley, Chengdu, Sichuan 610041 PR. China

**Keywords:** Elderly, Colorectal cancer, Adjuvant chemotherapy, Capecitabine, Low-dose, Non-inferiority, Randomized controlled trial

## Abstract

**Background:**

Over half of the patients were diagnosed with colorectal cancer after 70 years of age. The choice of the most suitable chemotherapy strategy is the major challenge for elderly patients. Previous trials indicated that elderly patients with stage II/III colorectal cancer obtained no significant benefits from oxaliplatin-based adjuvant chemotherapy. Therefore, single-agent oral capecitabine is regarded as an effective alternative with retained efficacy and improved flexibility. However, the optimal dose of capecitabine for elderly patients remains controversial. Recent studies have adopted a low-dose strategy (1,000 mg/m^2^) for elderly patients, but the long-term efficacy of this strategy has not been identified so far. Thus, we designed this trial to investigate non-inferiority of the lower-dose strategy of capecitabine compared with the approved-dose strategy for adjuvant chemotherapy of elderly patients with stage II/III colorectal cancer.

**Methods:**

LC-ACEC (Low-dose Capecitabine Adjuvant Chemotherapy for Elderly Patients With Stage II/III Colorectal Cancer) is a prospective, randomized, open-label, non-inferiority phase III clinical trial including 926 eligible patients. Patients will be randomly assigned to receive a capecitabine adjuvant chemotherapy strategy of lower dose (1,000 mg/m^2^ twice daily on days 1 to 14 of every 21 days) or approved dose (1,250 mg/m^2^ twice daily on days 1 to 14 of every 21 days). The primary outcome is 3-year disease-free survival. Secondary outcomes include 3-year overall survival, toxic and side effects during treatment, completion rate, and quality of life.

**Discussion:**

This is the first randomized trial to evaluate the efficacy and safety of a low-dose strategy of capecitabine in adjuvant chemotherapy of elderly patients with stage II/III colorectal cancer, and the results are believed to provide new evidence on the treatment of elderly patients with colorectal cancer.

**Trial registration:**

ClinicalTrials.gov identifier NCT02316535 (Dec. 12, 2014).

## Background

In Western countries, the incidence of colorectal cancer (CRC) peaked in patients more than 70 years old [1]. As for Chinese patients with CRC, the median age of diagnosis was 50 in the 1980s; however, it had reached 58 in 2005 and has been rising recently [2]. Limited by age-related organ function decline and comorbid conditions, elderly patients show impaired physical and mental tolerance for cancer treatment, such as chemotherapy.

Generally, stage II (T_3-4_N_0_M_0_) and stage III (T_any_N_1-2_ M_0_) colorectal carcinoma, the most common CRC in Chinese patients [3], can be resected by surgery. Additional adjuvant chemotherapy could significantly improve survival of stage II/III CRC patients compared with surgery alone [4]. Results of a pooled analysis including 3,351 CRC cases indicated that, in view of both efficacy and safety, elderly patients (more than 70 years old) achieved the same benefits from adjuvant chemotherapy as younger patients [5]. According to the large-scale retrospective analysis of the Adjuvant Colon Cancer Endpoints (ACCENT) database, patients at least 70 years of age accounted for only 17.1 % of all cases [6], which was much lower than expected. Therefore, improving the acceptance and completion rate of adjuvant chemotherapy for elderly patients with stage II/III CRC has become imperative.

The oxaliplatin-based combination chemotherapy (Folfox, Xelox) has been widely recommended recently [7]. However, the ACCENT analysis reported that adding oxaliplatin extended no significant benefits for elderly patients [6], suggesting that single-agent chemotherapy might be preferred for those patients. Capecitabine (Xeloda; Hoffmann-La Roche Inc., Nutley, NJ, USA), as the oral prodrug of 5-fluorouracil (Fu) recommended by global guidelines [8–10], has been proven to be at least equivalent to Fu bolus and intravenous Fu plus leucovorin (Lv) adjuvant chemotherapy for CRC [11–13] and to haveenhanced flexibility and compliance [12, 14]. Currently, the approved dose of capecitabine is 1,250 mg/m^2^ twice a day (bid) on days 1 to 14 of every 21 days [15], but the optimal dose for various groups of patients characterized by different ethnicity and age remains controversial. Moreover, although only relatively healthy elderly patients—that is, age of not more than 75 years and Eastern Cooperative Oncology Group (ECOG) performance status (PS) of not more than 1—were eventually included, the X-ACT (Xeloda in Adjuvant Colon Cancer Therapy) trial (m66001) found that 51 % of those patients undergoing 1,250 mg/m^2^ strategy required dose reduction to complete the whole chemotherapy, significantly more than younger patients, indicating that the approved dosage might be an overdose for elderly patients [16]. A previous non-randomized comparative analysis by Bajetta *et al*. reported that the completion rate (with no dose reduction) of a low-dose strategy of capecitabine (1,000 mg/m^2^ bid) was higher than the approved strategy (95 % versus 70 %), further concluding that 1,000 mg/m^2^ could be applied as a new recommended strategy for elderly patients with breast cancer [17]. The AVEX (bevacizumab plus capecitabine versus capecitabine alone in elderly patients with previously untreated metastatic colorectal cancer) trial (ClinicalTrials.gov identifier NCT00484939) in 2013 adopted the 1,000 mg/m^2^ low-dose strategy for metastatic CRC patients at least 70 years old [18]. However, it is well established that patients of different tumor stage have significantly different clinical characteristics. A recent single-arm study has verified feasibility of this starting dose at 1,000 mg/m^2^ bid for elderly (at least 70 years old) patients with stage II/III colon cancer [19] but has not produced results of efficacy. Thus far, however, no data on the optimal dose of capecitabine applied in elderly patients with stage II/III CRC have been available. Furthermore, the long-term efficacy of this 1,000 mg/m^2^ strategy, though widely used in elderly patients with cancer, has not been identified in comparison with the approved strategy.

For more individualized evidence guiding CRC treatment, we designed a non-inferiority randomized trial to identify the efficacy and safety of the low-dose capecitabine strategy (1,000 mg/m^2^ bid) for adjuvant chemotherapy of elderly (at least 70 years old) patients with stage II/III CRC.

## Methods

### Study objective

The primary objective of the LC-ACEC (Low-dose Capecitabine Adjuvant Chemotherapy for Elderly Patients With Stage II/III Colorectal Cancer) trial is to investigate the non-inferiority of the efficacy of low-dose capecitabine (1,000 mg/m^2^ bid) applied in adjuvant mono-chemotherapy for elderly patients with stage II/III CRC. Furthermore, we aim to compare the toxic and side effects, completion rate, and quality of life (QoL) of the low-dose strategy with the approved-dose strategy (1,250 mg/m^2^ bid).

### Study design and setting

**Fig. 1:**
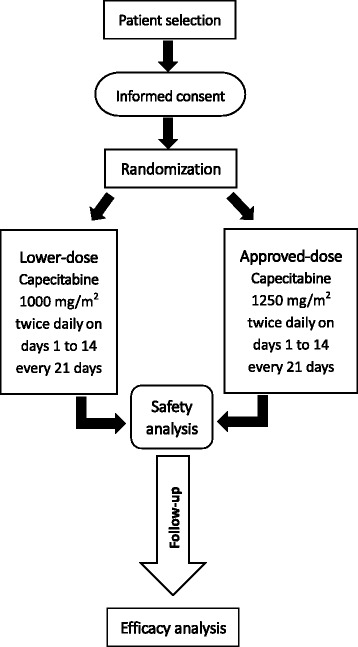
Overall flowchart of the LC-ACEC (Low-dose Capecitabine Adjuvant Chemotherapy for Elderly Patients With Stage II/III Colorectal Cancer) trial

This is a prospective, randomized, open-label, non-inferiority phase III clinical trial with two parallel groups. Patients in the low-dose group receive 1,000 mg/m^2^ bid capecitabine postoperatively; accordingly, patients in the approved-dose group receive 1,250 mg/m^2^ bid capecitabine. Fig. 1 illustrates the overall flow of participants in this trial.

Our trial began in January 2015 in the West China Hospital (WCH) of Sichuan University in China and is expected to end in 2023.

### Ethical considerations

This trial protocol is approved by the Biological and Medical Ethics Committee of WCH (reviewed in 2014 as #74) and has been registered at ClinicalTrials.gov (identifier NCT02316535). All of the eligible participants and their legal surrogates will be fully informed of potential risks and benefits of interventions in each group; only patients who are willing to provide written informed consent can be enrolled in the trial. The results of the trial will be reported in accordance with the CONSORT (Consolidated Standards of Reporting Trials) statement.

### Study population

The selection criteria of our trial are as follows:

#### Inclusion criteria

1. With written informed consent
2. Pathologically identified carcinoma of colon and rectum
3. pT3-4NanyM0 or pTanyN + M0 (stage II/III)
4. Age of at least 70 years to not more than 90 years
5. Underwent R0 resection (that is, no residue tumor)
6. ECOG PS of not more than 2
7. Life expectancy of at least 3 months
8. Initiation of adjuvant therapy within 12 weeks after surgery [20].

#### Exclusion criteria

1. With other types of malignances within the past 5 years
2. Creatinine clearance of not more than 50 mL/minute
3. History of neoadjuvant chemotherapy
4. In anticoagulant therapy
5. History of angina pectoris, congestive heart failure, or myocardial infarction within the past 12 months
6. History of other antineoplastic drugs
7. History of substance abuse
8. With other contraindications for chemotherapy (for example, thrombocytopenia, ongoing infection, and impaired liver function).

### Randomization and blinding

All participants fulfilling the inclusion criteria will be randomly allocated into either the low-dose or the approved-dose group. We use a computer-generated blocked randomization sequence with the block size of four according to the previous published trial conducted in WCH [21]. Patients are randomly assigned by stratification of disease stage (stage II or III) and primary tumor site (colon or rectum). To achieve allocation concealment, opaque envelopes with sequential numbers are adopted to seal the assignment and are accessible only for investigators not involved with this trial. After enrollment of each patient, the envelope is sent to an independent investigator, who is not involved in patient evaluation, to assign participants to either the low-dose or the approved-dose group. This is an open-label trial; thus, patients, investigators, and data analysts are aware of the group assignment.

### Intervention

About 4 to 8 weeks after surgery [20], patients in the low-dose group will take oral capecitabine after breakfast and dinner at the dose of 1,000 mg/m^2^ bid, and patients in the approved-dose group will receive the dose of 1,250 mg/m^2^ bid. Each treatment cycle consists of 14 days of capecitabine administration and then 7 days of rest.

**Fig. 2:**
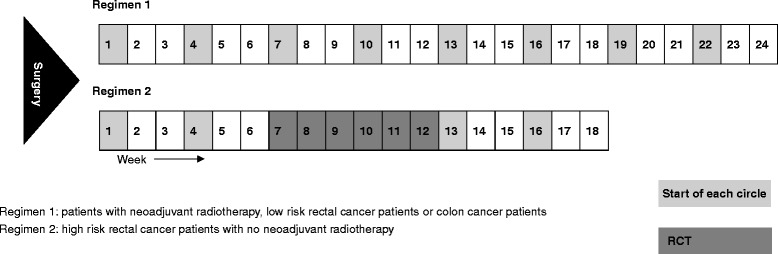
Treatment regimen. RCT, radiochemotherapy

Colon cancer patients in both groups are supposed to take eight circles of treatment in total (24 weeks) [22]. Eight circles of treatment will also apply to rectal cancer patients who had neoadjuvant radiotherapy (regimen 1 in Fig. 2). For rectal cancer patients without neoadjuvant radiotherapy, we defined high-risk patients who need concomitant radiochemotherapy (RCT) as patients with positive circumferential resection margin (CRM+) or with lymph node metastasis (N+), and they will receive a total of five circles of chemotherapy [13]. The RCT serves as the third circle and lasts about 6 weeks [13]. During the RCT period, the irradiation dose is 45 Gy/25 fractions, and the capecitabine dose is reduced to 800 mg/m^2^ bid [23] (regimen 2 in Fig. 2). According to the toxic events, the dose of capecitabine will be adjusted following the drug instruction of Xeloda® [15]. Any patients who have intolerable toxic or side effects will discontinue the medication.

### Outcomes

#### Primary outcome

The primary outcome of the LC-ACEC trial is 3-year disease-free survival (DFS), which is calculated from the date of randomization to whichever of the following events occurs first in 3 years: local recurrence, metastasis, death from any causes, or development of other malignances.

#### Secondary outcome

Several secondary outcomes are adopted in this trial. In addition to the primary outcome of 3-year DFS, the 3-year overall survival is calculated from the randomization to the date of death from any causes. Toxic and side effects during adjuvant chemotherapy will be closely observed and evaluated by using National Cancer Institute Common Toxicity Criteria (NCI-CTC) version 2.0. Based on the toxic and side effects, we will keep every record of modification or discontinuance of capecitabine and calculate the completion rate of the treatment as one of the key secondary outcomes. Moreover, QoL data are recorded by using a Chinese translation of European Organisation for Research and Treatment of Cancer Core QoL Questionnaire C30 (EORTC QLQ-C30) [24].

#### Follow-up and data collection

The medical history and physical examination will be taken immediately at the outpatient clinic. After patients pass the selection procedure and sign the informed consent form, a series of investigations, including complete blood test (for example, blood cell count and blood chemistry), tumor markers of carcinoembryonic antigen (CEA) and carbohydrate antigen 19-9 (CA 19-9), total abdominal computed tomography (CT) scan, and chest radiograph, will be scheduled. It is worth mentioning that before every circle of chemotherapy, renal function of each participant will be monitored and evaluated by calculating the creatinine clearance. When participants finish every circle of chemotherapy, investigators will assess the toxic and side effects by using NCI-CTC and record any modification or discontinuation of capecitabine. In view of possible compromised compliance of elderly patients, returned medication will be checked at the clinic, and patients who discontinue the treatment for more than 1 week (except for toxic effects) will be withdrawn from this trial. When the whole treatment is over, patients will come to the hospital for a check-up every 3 months in the first 2 years and every 6 months afterward. Each time, the complete blood test, tumor markers, total abdominal CT scan, chest radiograph, and rectal examination will be conducted to help oncologists verify the possible local recurrence and metastasis, and the QoL questionnaire will be filled. We will follow up patients on a corresponding timeline at the clinic or by phone. All the data of each patient will be recorded in a case report form (CRF) in time.

#### Safety and data monitoring

An independent data and safety monitoring board (DSMB) composed of oncologists, gastrointestinal surgeons, and statisticians is responsible for reviewing trial data, including adverse events (AEs) and drop-outs, at least twice a year. If any unexpected AEs happen or any types of AEs occur more frequently than our own Chinese database and most authors reported, members of the DSMB will consider early closure of this trial according to relevant Chinese laws and regulations. AEs in this trial are evaluated by using NCI-CTC as a secondary outcome. Severe adverse events (SAEs) in this trial include death, disability, and prolonged hospitalization. Both AEs and SAEs will be recorded in CRFs and the DSMB will be informed within 24 hours.

#### Sample size and statistics

The LC-ACEC trial aims to identify the non-inferiority of 3-year DFS of patients in the low-dose capecitabine adjuvant chemotherapy group versus the approved-dose group. We assumed that a background 3-year DFS was 60 % and predefined the non-inferiority margin of the hazard ratio (HR) as 1.30. When 456 events of the primary endpoint 3-year DFS occur, the efficacy analysis will be conducted. We chose a statistical power of 0.80 and a significance level of 0.05 (two-sided). Given the 10 % drop-out for elderly patients, the calculation yielded a sample size of 926 patients (463 in each arm) [11, 25].

The intention-to-treat analysis will be applied with all patients who undergo randomization in this trial. With regard to survival data, HRs with 95 % confidence intervals (CIs) will be presented by Kaplan-Meier survival estimates. Additionally, Cox proportional HRs with 95 % CIs will be calculated on the basis of subgroups of different characteristics (that is, age, sex, stratum, and Charlson comorbidity index), which can identify the influence of competing risk factors for the outcome event. For the primary outcome of 3-year DFS, the non-inferiority will be concluded if the upper limit of the 95 % CI is not more than 1.30 (predefined non-inferiority margin). As for other outcomes, proportions will be compared by using chi-squared test, and continuous data will be analyzed by *t* test. All the statistical analyses will be conducted by using SPSS version 20 software (SPSS, Inc., Chicago, IL, USA).

## Discussion

It is well established that age is a major risk factor for CRC. According to available statistics, over half of patients with CRC are at least 70 years old, and approximately 43 % of them are at least 75 years old [26]. For stage II/III CRC, adjuvant chemotherapy has been demonstrated to significantly improve survival, and these benefits are not influenced by age [5]. Therefore, there is no doubt that adjuvant chemotherapy should be applied for elderly patients with stage II/III CRC. In reality, however, elderly patients are generally undertreated with chemotherapy because of relatively poor health, and this resulted in a lower participation rate of elderly patients in previous large-scale studies [6] and compromised applicability of those conclusions to elderly patients. Currently, relatively little is known about adjuvant chemotherapy specifically for elderly patients with stage II/III CRC.

The fact that elderly patients obtained no significant benefits from adding oxaliplatin indicated that singe-agent chemotherapy could apply to elderly patients [6]. Compared with intravenous chemotherapy of 5-Fu/Lv, oral administration of capecitabine can remarkably enhance flexibility and compliance, contributing to a higher participation rate for the elderly patients. The major challenge, especially for elderly patients, is to balance the survival benefits and impaired QoL caused by toxic and side effects. Considering the final results of the X-ACT trial, Twelves *et al*. [16] found that patients who experienced fewer toxic and side effects had worse outcomes, implying that dose reduction of capecitabine might compromise the efficacy. Thus far, no published data have identified the long-term efficacy and tolerance of the low-dose strategy.

For capecitabine, the dose of 1,000 mg/m^2^ bid is prescribed frequently in clinical practice and has been reported to be active and well tolerated among elderly patients with breast cancer [17, 27]. Although the response rate was assessed, no long-term results of survival data have been reported for those patients with breast cancer. As for CRC, the low-dose strategy was used in the AVEX trial (ClinicalTrials.gov identifier NCT00484939) for elderly patients with metastatic CRC; however, no comparative analysis about long-time efficacy of this strategy compared with the approved strategy has been published so far. Considering the current gap, we designed this LC-ACEC trial. If our results find compromised long-term efficacy of the low-dose strategy, it might not be an optimal dose for elderly patients with stage II/III CRC although it is widely used at present.

Notably, previous studies reported ethnic differences in the tolerance of capecitabine. Haller *et al*. [28] found that East Asian patients had better tolerance of 5-Fu/capecitabine compared with Caucasian patients. An early study indicated that the thymidylate synthase (*TYMS*) gene 3R/3R variants were more common in Asians [29], and this could affect 5-Fu tolerance. For the elderly Asian patients, however, the pilot Korean study did not observe a satisfactory completion rate for the 1,250 mg/m^2^ dose despite better tolerance for Asians [19]. As for China, a single-arm study including 58 elderly (more than 65 years old) patients with metastatic CRC showed that low-dose capecitabine was well tolerated by those patients [30]. However, that study was limited by the lack of data regarding efficacy and the small sample size. In general, current evidence on the dose of capecitabine for the elderly group of patients is insufficient.

It is worth mentioning that our trial still has limitations. The blinding is limited by the open-label nature of the trial. In addition, time of follow-up is relatively short to achieve long-term outcome given the loss rate caused by high population mobility in this region. Moreover, our trial includes only Chinese patients and this to some degree limits the applicability to other ethnicities because of inter-ethnic differences in tolerance for capecitabine. In summary, the LC-ACEC trial is the first randomized controlled trial to investigate the efficacy and safety of the low-dose capecitabine adjuvant chemotherapy (1,000 mg/m^2^ bid) in elderly patients with stage II/III CRC. If non-inferiority of the low-dose strategy is verified, this can be recommended as a new standard capecitabine adjuvant chemotherapy strategy, specifically for the large group of elderly patients with stage II/III CRC, and this is not only cost-effective but also beneficial for their QoL. The result of this trial will provide evidence on a more specific adjuvant chemotherapy strategy for elderly patients with stage II/III CRC.

## Trial status

This trial was initiated in January 2015 and is currently recruiting patients.

## Abbreviations

ACCENT: Adjuvant Colon Cancer Endpoints
AE: Adverse event
AVEX: bevacizumab plus capecitabine versus capecitabine alone in elderly patients with previously untreated metastatic colorectal cancer
bid: twice a day (*bis in die*)
CI: confidence interval
CRC: colorectal cancer
CRF: case report form
CT: computed tomography
DFS: disease-free survival
DSMB: data and safety monitoring board
ECOG: Eastern Cooperative Oncology Group
Fu: fluorouracil
HR: hazard ratio
LC-ACEC: Low-dose Capecitabine Adjuvant Chemotherapy for Elderly Patients With Stage II/III Colorectal Cancer
Lv: leucovorin
NCI-CTC: National Cancer Institute Common Toxicity Criteria
PS: performance status
QoL: quality of life
RCT: radiochemotherapy
SAE: severe adverse event
X-ACT: Xeloda in Adjuvant Colon Cancer Therapy
WCH: West China Hospital
